# On the Piezomagnetism of Magnetoactive Elastomeric Cylinders in Uniform Magnetic Fields: Height Modulation in the Vicinity of an Operating Point by Time-Harmonic Fields

**DOI:** 10.3390/polym16192706

**Published:** 2024-09-25

**Authors:** Gašper Glavan, Inna A. Belyaeva, Mikhail Shamonin

**Affiliations:** East Bavarian Centre for Intelligent Materials (EBACIM), Ostbayerische Technische Hochschule (OTH) Regensburg, Seybothstr. 2, 93053 Regensburg, Germany; inna.belyaeva@oth-regensburg.de (I.A.B.); mikhail.chamonine@oth-regensburg.de (M.S.)

**Keywords:** magnetoactive, magnetorheological, elastomer, strain, magnetostriction, piezomagnetic, bias field, macroscopic deformation, time-harmonic field

## Abstract

Soft magnetoactive elastomers (MAEs) are currently considered to be promising materials for actuators in soft robotics. Magnetically controlled actuators often operate in the vicinity of a bias point. Their dynamic properties can be characterized by the piezomagnetic strain coefficient, which is a ratio of the time-harmonic strain amplitude to the corresponding magnetic field strength. Herein, the dynamic strain response of a family of MAE cylinders to the time-harmonic (frequency of 0.1–2.5 Hz) magnetic fields of varying amplitude (12.5 kA/m–62.5 kA/m), superimposed on different bias magnetic fields (25–127 kA/m), is systematically investigated for the first time. Strain measurements are based on optical imaging with sub-pixel resolution. It is found that the dynamic strain response of MAEs is considerably different from that in conventional magnetostrictive polymer composites (MPCs), and it cannot be described by the effective piezomagnetic constant from the quasi-static measurements. The obtained maximum values of the piezomagnetic strain coefficient (∼$10^{2}$ nm/A) are one to two orders of magnitude higher than in conventional MPCs, but there is a significant phase lag (35–60°) in the magnetostrictive response with respect to an alternating magnetic field. The experimental dependencies of the characteristics of the alternating strain on the amplitude of the alternating field, bias field, oscillation frequency, and aspect ratio of cylinders are given for several representative examples. It is hypothesized that the main cause of observed peculiarities is the non-linear viscoelasticity of these composite materials.

## 1. Introduction

**Figure 1:**
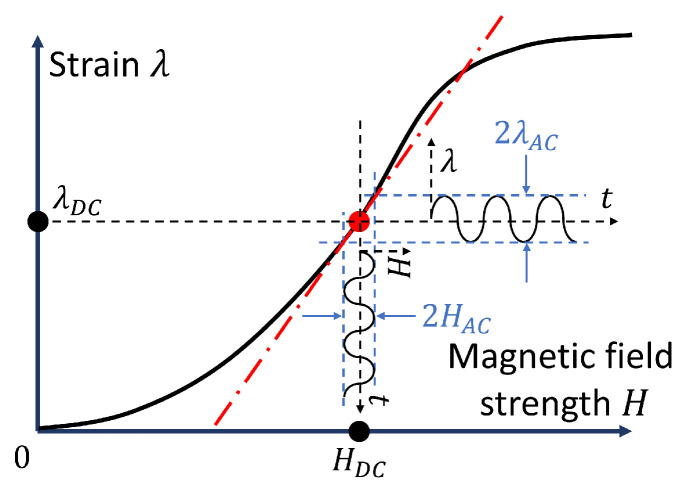
Schematic diagram of the dynamic behavior of a conventional MS actuator driven by a time-harmonic magnetic field in the vicinity of a bias point ($H_{DC},\lambda_{DC}$). The red dash-dotted line denotes the tangent of the curve λ(H) at point $H=H_{DC}$.

This paper concerns the concept of piezomagnetism (or linear magnetostriction) [1]. It is well known that the magnetostriction of conventional solid ferromagnetic materials is a second-order effect with respect to the applied magnetic field [2,3,4]. This means that the sign of the magnetostrictive (MS) strain does not change when the direction of the magnetic field strength is reversed. For example, a rod of a ferromagnetic material increases in length for both positive and negative values of the magnetic field strength. However, in conventional MS materials, magnetostriction “can be represented as effectively first-order when variations in the system parameters are small compared with the initial values of the parameters” [5]. Figure 1 illustrates the concept of the direct piezomagnetic effect in the context of employing an MS material as an actuator [5,6,7]. The effective piezomagnetic constant *d* can be calculated from the quasi-static field dependence of the magnetostrictive strain λ by calculating the first derivative of λ with respect to the applied magnetic field $H_{DC}$: $d=\partial \lambda /\partial H_{DC}$. Traditionally, it can be expected that the maximum piezomagnetic response is observed in the biased magnetic field, where the effective piezomagnetic constant *d* has its maximum.

For example, piezomagnetism is important for designing layered magnetoelectric (ME) sensors of AC magnetic fields comprising MS and piezoelectric layers [8,9,10,11,12,13]. In this context, the maximum ME voltage coefficient is expected in the bias magnetic field, where the magnitude of the effective piezomagnetic constant *d* of the MS layer has the highest value. Very recently, a new concept for designing layered multiferroic structures has been put forward in the literature [14,15,16,17]. The idea here is to depart from conventional rigid materials (ferromagnetic metals and alloys as well piezoelectric ceramics and single crystals) and to employ much softer polymer-based materials. The advantage of such an approach is the much lower electromechanical resonance frequency of the resulting heterostructures in comparison with conventional heterostructures [18,19]. The usage of polymer-based ferromagnetic and piezoelectric phases brings into play new physical effects related to their softness and viscoelasticity [20]. The ferromagnetic phase in layered polymer-based multiferroic heterostructures employed the magnetorheological or magnetoactive elastomers (MAEs), which consist of magnetic micro- or nanoparticles embedded into a polymer matrix [21,22,23,24,25,26,27,28].

However, our main motivation for investigating the piezomagnetic phenomena in MAEs is potential application of these materials in magnetically controlled soft actuators for soft robotics [29,30] owing to much higher deformations of MAEs (longitudinal strain up to $10^{-2}$–$10^{-1}$) in technically feasible magnetic fields as compared to conventional (pure metals and alloys) MS materials (strain up to about $2\times 10^{-3}$) [31,32,33,34,35,36,37,38,39,40,41,42,43].

Investigations of MS phenomena in composite ferromagnetic materials, which account for a strong viscoelastic response in the polymer matrix, are scarce and mostly of a theoretical nature [44,45,46]. It was reported that the inclusion of matrix viscosity results in an apparent hysteresis loop in magnetization and magnetostriction curves even though the model does not include magnetoelastic hysteresis in the magnetostrictive fibers [44]. Indeed, soft MAEs demonstrate a significant hysteresis of different physical properties, which is commonly attributed to the hysteresis of the consolidation of filler particles into elongated aggregates, i.e., the dependence of the internal microstructure of composite on the magnetization history [47,48,49]. In [50], it was pointed out that the dynamic behavior in certain scenarios of MAE-based actuators with soft-magnetic filling particles remains unexplored.

Hitherto, the effects of magnetic field variations around a biased magnetic field on magnetically induced strain in MAEs remain unstudied. Such a statement of the problem is similar to that of piezomagnetism in conventional ferromagnetic materials. The purpose of the present paper is to experimentally investigate the macroscopic deformation of MAE cylinders in the vicinity of an operation point upon superimposed time-harmonic magnetic-field excitation. The term time-harmonic indicates that only sinusoidal time-varying fields are considered [51]. The paper is organized as follows: The fabrication of MAE cylinders, the experimental setup, and the measurement protocol are described in Section 2. The results of measurements are presented and discussed in Section 3. In particular, the dependencies of the piezomagnetic strain coefficient $d_{33}$ and the phase lag ϕ of the time-harmonic strain with respect to the time-harmonic magnetic field on the biased magnetic field, the cylinder’s aspect ratio, the amplitude of the AC field, and its frequency are presented for the first time.

## 2. Materials and Methods

### 2.1. Synthesis of MAE Cylinders

This article is a follow-up paper to the research commenced in [41,52], where the fabrication of MAE materials and their characterization were described in detail. Below, we recall the main points. MAE composites were based on one and the same polydimethylsiloxane (PDMS) matrix with a stoichiometry ratio of the reaction (the ratio of the molar concentrations of hydride and vinyl reactive groups) r≈1 [41,52]. In the theoretical case, there should be only elastically active chains in the polymer network [53]. Carbonyl iron powder (CIP, type SQ, mean particle size of 3.9–5.0 μm, BASF SE, Ludwigshafen, Germany) was used as a soft-magnetic filling. The routine for synthesizing MAE materials was previously described in [54,55]. Liquid compound mixtures were poured into 3D printed moulds from a high-temperature-resistant material, acrylonitrile butadiene styrene (ABS) [41,52]. The curing took place in an oven with air circulation (Memmert model UF30, Memmert GmbH + Co. KG, Schwabach, Germany) for 1 h at 80 °C and next 24 h at 60 °C.

Cylinders differed in the anisotropy of particle arrangement (isotropic/anisotropic), the concentration of iron particles (70, 75 and 80 wt%, corresponding to three volume fractions of 22, 27 and 33 vol%), and the aspect ratio $\Gamma_{0}=h_{0}/D_{0}$ (0.2, 0.4, 0.6, 0.8, 1.0 and 1.2), where the initial diameter $D_{0}$ was kept constant at 15 mm and the initial cylinder height $h_{0}$ was varied. Isotropic cylinders were cured in the absence of a magnetic field. To obtain anisotropic samples, two permanent samarium cobalt (SmCo) magnets (diameter of 25 mm and thickness of 5 mm, magnetized along the axis of the cylinder) were placed on the sides of the filled moulds [41,52].

Reference samples were produced for each material composition to analyze the rheological properties of MAE materials. Rheological measurement was conducted with a commercial rheometer (Anton Paar, model Physica MCR 301, with a magnetorheological device (MRD301) in the plate–plate (PP20/MRD/TI) geometry. The angular frequency ω=10 s^−1^ and the shear oscillation amplitude γ=0.01% were constant through all the measurements. The results obtained and the chemical compositions are described in detail in [41]. The shear storage modulus of the PDMS matrix was about 7.7 kPa, and the shear loss modulus was roughly 0.3 kPa.

Magnetization curves of similar MAE materials have been published elsewhere [56]. Magnetization curves demonstrated a typical “pinched” hysteresis loop behavior. In comparison to magnetostrictive hysteresis loops, the magnetic hysteresis is barely pronounced. This means that the ascending and descending branches of magnetization versus external magnetic field were quite close to each other [56].

### 2.2. Experimental Setup

**Figure 2:**
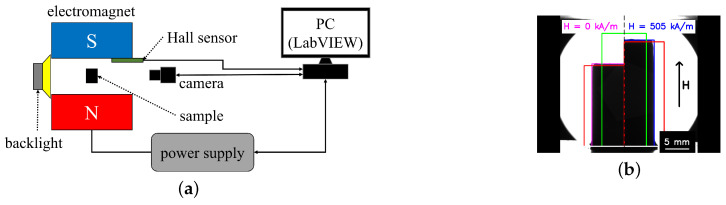
(**a**) Schematic diagram of the experimental setup. (**b**) Images of an isotropic MAE cylinder with an iron content 75 wt% and an aspect ratio $\Gamma_{0}$ of 1.2 in a zero magnetic field (**left** side) and in a maximum magnetic field (**right** side).

MAE cylinders were attached to a 3D printed holder made from polylactic acid (PLA) and positioned between the poles of an electromagnet (EM2 model, MAGMESS Magnetmesstechnik Jürgen Ballanyi e.K., Bochum, Germany), powered by a bi-polar power supply (FAST-PS 1k5, CAENels s.r.l., Basovizza, Italy). The magnetic field generated is known to possess high uniformity [56]. The deformation of the cylinders was recorded in side view using a CMOS camera (Alvium 1800 U-319 m, Allied Vision Technologies GmbH, Stadtroda, Germany). The experimental setup (Figure 2a) was enhanced in comparison with the previous works [41,52]. A lens with the decreased view area and thus increased accuracy of measurements (Kowa LM35JC10M 2/3″ 35 mm/F2.0 C-Mount, Kowa Optimed Deutschland GmbH, Düsseldorf, Germany) was employed. The cylinders were backlit by a light-emitting diode (LED; Illuminant G4 Pen, Conrad Electronics, Hirschau, Germany) through a diffuser (Perspex diffuse, 2.5 mm, 3A Composites GmbH, Sins, Switzerland). The cylindrical samples were placed vertically between the electromagnet poles so that the cylinder axis was parallel to the magnetic field lines. The camera’s vertical position was adjusted each time to maintain a constant angle between the camera and the top edge of a cylinder. The experiment was automated using LabVIEW software (version 2018, National Instruments, Austin, TX, USA) [41,52].

### 2.3. Image Processing

An example of obtained images is shown in Figure 2b, which compares images of an isotropic MAE cylinder in a zero magnetic field (left side of the figure) and a maximum magnetic field (right side of the figure). The left side of Figure 2b shows half of the cylinder in a zero magnetic field. Shown is a fragment of the entire image, which is cut along the cylinder´s axis. Similarly, the right side displays the image of the other half of the same cylinder in the maximum magnetic field. The changes in the cylinders’ dimensions were obtained using the sub-pixel edge algorithm from [57], implemented in Python 3.12 The contact plane, indicated with the white horizontal line, was determined manually. The cylinder´s height *h* was determined inside a rectangular region of interest (ROI) with a width of 500 px as the difference of an average horizontal coordinate (*x*) of the top edge obtained with a sub-pixel edge detection algorithm and the manually determined contact plane. This ROI is shown as a green rectangle in Figure 2b. The pixel size was approximately 19×19 μm. According to [57], the uncertainty of edge detection was about ±0.04 px. Two red ROIs (Figure 2b) were used to obtain the left and right edges of the cylinder. The differences between the averages of vertical coordinates (*y*) of these two edges return cylinders with a diameter of *D*. Dimensions of these two ROIs vary with the cylinders’ deformation in such a way that the top edge is always 30 px below the calculated top edge obtained from green ROI. The magenta and blue lines on the edge of the MAE cylinder mark the calculated edges of the cylinder by the just-described algorithm. Apparent longitudinal strains λ were calculated from height changes: $\lambda =\left(h-h_{0}\right)/h_{0}$ [41,52].

### 2.4. Measurement Protocol

**Figure 3:**
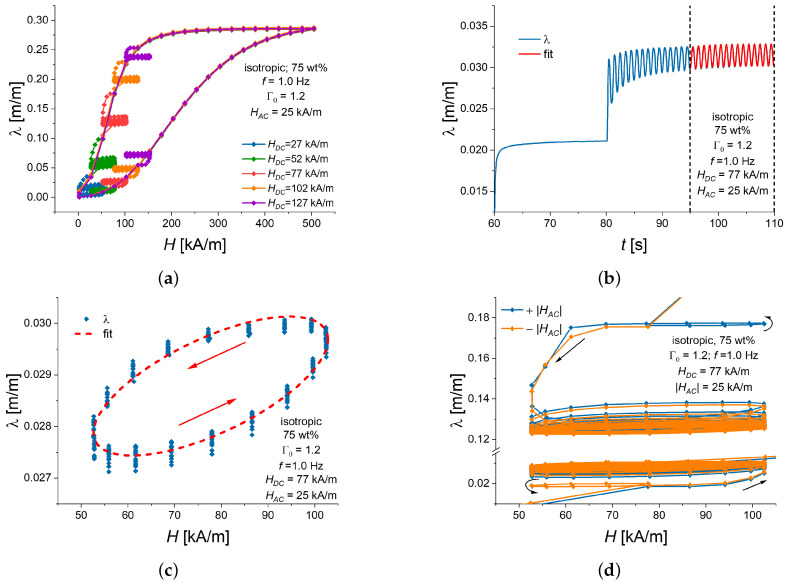
Macroscopic deformation of an isotropic MAE cylinder with 75 wt% of Fe and an aspect ratio $\Gamma_{0}=1.2$. Here and below, a line connecting experimental points serves as a guide to the eye. (**a**) Magnetostrictive hysteresis loops with additional minor loops due to superimposed oscillations of a magnetic field at different operation points. (**b**) Example of the transient response of magnetostrictive strain in a magnetic field (1). The measured values are shown in blue, and the fitted sinusoidal function is shown as a red curve. The bias field of 77 kA/m was set at the time point of t=60 s. (**c**) Longitudinal strain λ versus the momentary value of magnetic field for the case (**b**). The red ellipse demonstrates the result from the fitted function (2). (**d**) Comparison of magnetostrictive response when magnetic field oscillations started with opposite phases ($H_{AC}$ had the same magnitude, but it was either positive or negative). In (**b**–**d**), magnetic field oscillations had a frequency *f* of 1.0 Hz, an oscillation amplitude $H_{AC}$ was 25 kA/m, and a bias magnetic field $H_{DC}$ was 77 kA/m.

Figure 3a shows the measured dependencies of the longitudinal strain on the applied magnetic field strength *H* for an MAE cylinder. Typical magnetostrictive loops were observed. The magnetic field was gradually increased from zero to the maximum value of 500 kA/m with a step of 25 kA/m and then gradually decreased to zero. Each value of the magnetic field *H* was kept constant for 20 s, so that the steady state deformation of the cylinder could be reached [52]. Once the desired bias magnetic field (operating point) $H=H_{DC}$ was arrived at, after 20 s, the magnetic field was varied with time as

(1) $$H=H_{DC}+H_{∼}\left(t\right)=H_{DC}+H_{AC}\cos\left(2\pi ft\right),$$

where $H_{∼}$ is the alternating magnetic field, $H_{AC}$ is the oscillation amplitude, and *f* is the oscillation frequency. As an example, Figure 3b presents the resulting variation in the longitudinal strain with time. The duration of magnetic field oscillations was fixed at 30 s. The total duration of measurements at an operating point was, therefore, 50 s. The sampling rate of images in the course of magnetic field oscillations was 18 fps. The bias magnetic field $H_{DC}$ was varied between 27 and 127 kA/m with a step of 25 kA/m, the oscillation amplitude of magnetic field $H_{AC}$ was between 15 and 65 kA/m with a step of 12.5 kA/m, and the oscillation frequency *f* took six different values between 0.1 and 2.5 Hz (0.1, 0.5, 1.0, 1.5, 2.0 and 2.5 Hz). Note that the same value of a bias magnetic field (operating point) $H_{DC}$ was set twice, for both ascending (increasing) and descending (decreasing) branches of the MS hysteresis loop. To ensure good repeatability and the same initial conditions, we allowed at least 24 h for the relaxation of samples between the measurements.

As an example, Figure 3b depicts the time response of the longitudinal strain to the applied oscillatory magnetic field. Some additional transient behavior is visible. To eliminate this transient effect, a sinusoidal function was fitted to the cylinder’s magnetostrictive response during the last 15 s of magnetic field oscillations. The fitted sinusoidal function can be written as

(2) $$\lambda =\lambda_{slow}\left(t\right)+\lambda_{∼}\left(t\right)=\lambda_{slow}\left(t\right)+\lambda_{AC}\cos\left(2\pi ft-\phi \right),$$

where $\lambda_{slow}$ is the transient longitudinal strain due to the step-like change in the bias field $H_{DC}$, $\lambda_{∼}$ is the alternating longitudinal strain, $\lambda_{AC}$ is the amplitude of the oscillatory response, and ϕ is the phase difference between the AC excitation field and the AC longitudinal strain. $\lambda_{slow}\left(t\right)$ was approximated as an exponential function $\lambda_{slow}\left(t\right)=\lambda_{1}+\Delta \lambda \left(1-e^{-at}\right)$, with $\lambda_{1}>0$, Δλ≶0 and a>0 as fitting constants. The actual oscillation frequency *f* was determined by fitting a sinusoidal function onto the measured oscillation of a magnetic field. Examples of more detailed transient responses of the longitudinal strain to a step-like change in the DC magnetic field and a superimposed time-harmonic field are presented in Appendix A.

When a sinusoidal oscillation of the longitudinal strain (2) is plotted against the sinusoidal magnetic field (1), a Lissajous figure in the form of an ellipse is produced. Figure 3c presents a comparison between measured values and fitted functions. It is seen that the measured values fit the elliptic shape very well. The rotation direction of the point on a Lissajous ellipse is counterclockwise. This is to be expected because the longitudinal strain lags after the magnetic field [58]. Small experimental deviations of experimental points from the fitted ellipsoidal shape of the resulting loop in Figure 3c can be attributed to the transient response of magnetostriction to time-varying magnetic field [52] and the uncertainty of measurements. In experiments, it was not possible to keep the amplitude $H_{AC}$ much smaller than $H_{DC}$ because of the challenge of measuring small variations of the resulting strain due to the limited spatial resolution of the camera. In what follows, we will analyze fitted Lissajous ellipses without offset values due to the presence of $H_{DC}$. This means that $\lambda_{∼}$ as functions of $H_{∼}$ will be presented.

**Figure A2:**
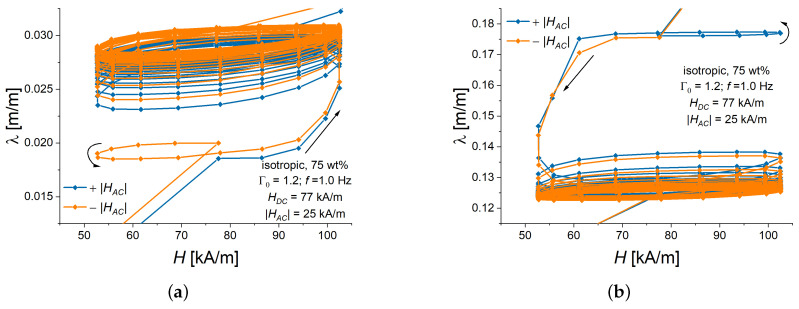
Magnetostrictive response to oscillating magnetic field when the bias magnetic field is increasing (**a**) and when the bias field is decreasing (**b**). Blue dots and lines refer to the positive value of $H_{AC}=25$ kA/m, and orange dots and lines refer to the negative value of $H_{AC}=-25$ kA/m. Parameters of the sample and measurement conditions are given within the figures.

As an example, Figure 3d shows the magnetostrictive response of an MAE cylinder when the field oscillations have a positive or negative sign of $H_{AC}$ in Equation (1). This means that a soft-magnetic MAE is insensitive to the direction of the AC magnetic field at the starting point and “forgets” minor details when attaining a steady cycle. It is visible that, although some differences did exist during the initial transient response of the longitudinal strain, the resulting Lissajous figures are practically the same. Investigations using the function (1) were sufficient to describe the relationships between the AC magnetic field and the AC longitudinal strain. For the sake of convenience for readers, Figure 3d is provided in more detail in Appendix B (Figure A2).

According to [59], the piezomagnetic strain coefficient,

(3) $$d_{33}=\lambda_{AC}⁄H_{AC},$$

can be used to characterize the dynamic properties of magnetostrictive polymer composites.

## 3. Results and Discussion

### 3.1. Dependence of the Alternating Strain on the Magnitude of $H_{AC}$

Figure 3b demonstrates that the magnetostrictive response of an MAE cylinder to a time-harmonic magnetic field $H_{∼}$ in the vicinity of a bias field $H_{DC}$ does resemble piezomagnetic behavior in conventional MS materials because there are positive and negative half waves of the longitudinal strain $\lambda_{∼}$. However, the strain response is delayed with regard to the alternating magnetic field. This can be explained by the viscoelasticity of a polymer matrix and the entire composite material.

**Figure 4:**
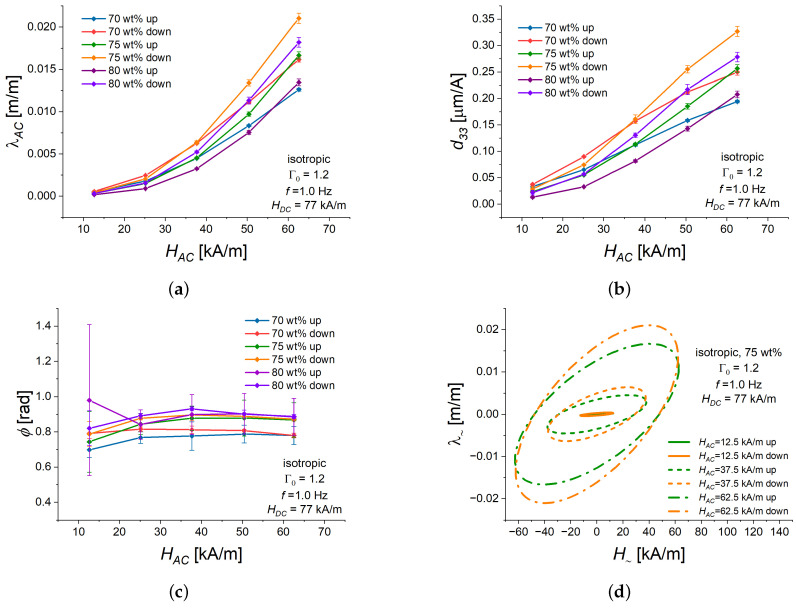
Dependencies of different characteristics of the alternating strain $\lambda_{∼}$ with a frequency *f* = 1.0 Hz on the magnitude of $H_{AC}$ for isotropic MAE cylinders with three different weight fractions of Fe (70, 75, 80 wt%) and $\Gamma_{0}=1.2$ in ascending (“up”) or descending (“down”) bias field $H_{DC}=77$ kA/m of a quasi-static magnetostrictive hysteresis loop. In all sub-figures, a specific line color refers to the same value of iron content and the same part of hysteresis loop (ascending/descending). (**a**) The amplitude of harmonic strain oscillation $\lambda_{AC}$ versus $H_{AC}$. (**b**) Piezomagnetic coefficient $d_{33}$ versus $H_{AC}$. (**c**) Phase lag ϕ versus $H_{AC}$. (**d**) Lissajous figures for different values of $H_{AC}$. Continuous lines refer to $H_{AC}=12.5$ kA/m, dashed lines refer to $H_{AC}=37.5$ kA/m, and dash-dotted lines refer to $H_{AC}=62.5$ kA/m.

Furthermore, in the investigated range of the amplitude $H_{AC}$ between 12.5 kA/m up to 65 kA/m in steps of 12.5 kA/m, the amplitude of alternating strain $\lambda_{∼}$ depends on the amplitude of $H_{AC}$ non-linearly. An example is shown in Figure 4a. For all isotropic samples with an aspect ratio of $\Gamma_{0}=1.2$, $\lambda_{AC}$ monotonically increased with increasing amplitude of the oscillatory magnetic field. The resulting piezomagnetic coefficients $d_{33}$ also monotonically increased with increasing $H_{AC}$ (Figure 4b). One of the reasons for the non-linear dependence of $\lambda_{AC}$ on $\left(H_{AC}\right)$ could be the Payne effect, which is characteristic of rubber composites containing fillers [60]. The Payne effect describes the decrease in the complex modulus of a filled, cross-linked elastomer system with increasing deformation amplitude. In MAEs, the Payne effect is also known to become well pronounced in magnetic fields, cf. [61,62,63] or Figure 1c,d in the very recent paper [50]. For MAEs considered in the present paper, an example of the Payne effect was given in Figure 4 of Ref. [41]. The highest sensitivity of $\lambda_{AC}$ to $H_{AC}$ was achieved for the sample with 75 wt of Fe, which could be expected from the previous investigation [41]. In general, for otherwise equal experimental parameters, $d_{33}$ was somewhat lower for the ascending bias field than for the descending bias field. This also could be expected from the higher piezomagnetic constant *d* for the descending part of the MS hysteresis loop than for the ascending part of the loop at the same value of the bias field.

The phase lag ϕ was weakly dependent on $H_{AC}$ (Figure 4c) and had a tendency to moderately increase with increasing $H_{AC}$. The dependencies of ϕ on $H_{AC}$ for samples with 80 and 75 wt% of Fe were within the uncertainty of measurements, while $\phi (H_{AC})$ for a cylinder with 70 wt% of Fe was about 0.1 rad lower. Figure 4d depicts Lissajous curves for the MAE cylinder with 75% of iron content and three representative values of $H_{AC}$ (lowest, highest, and medium value). The features of the magneto-deformation response described above to the alternating magnetic field are clearly visible there.

The following results are presented for the fixed value $H_{AC}=25$ kA/m. We have selected it as a compromise between a sufficiently high value of $H_{AC}$ for the accurate measurements of AC strain and the increasing non-linearity of the piezomagnetic response with the increase in $H_{AC}$. As examples, we used isotropic MAE cylinders with 75 wt% of Fe because the samples with this filler concentration demonstrated the highest MS response in quasi-static experiments [41]. The aspect ratio $\Gamma_{0}$ of 1.2 was employed, since it resulted in the highest absolute elongation in quasi-static experiments [41].

### 3.2. Dependence of the Alternating Strain on the Bias Magnetic Field

**Figure 5:**
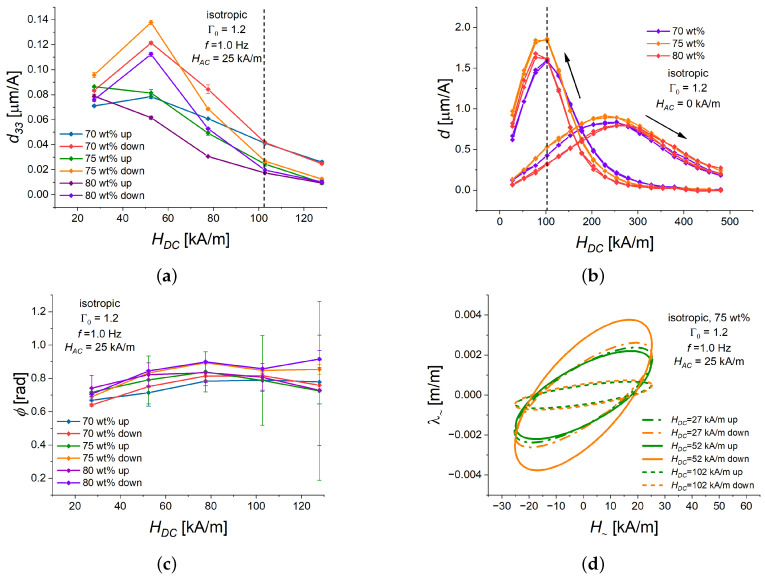
Dependencies of different characteristics of the alternating strain $\lambda_{∼}$ with a frequency *f* = 1.0 Hz on the magnitude of $H_{DC}$ for isotropic MAE cylinders with three different weight fractions of Fe (70, 75, 80 wt%), and $\Gamma_{0}=1.2$ in the ascending (“up”) or descending (“down”) bias field $H_{DC}$ of a quasi-static magnetostrictive hysteresis loop. The value of $H_{AC}$ is fixed at 25 kA/m. In all sub-figures, a specific line color refers to the same value of iron content and the same part of hysteresis loop (ascending/descending). (**a**) $d_{33}$ versus $H_{DC}$. Dashed black line designates the position of the maximums of *d* for the descending part of the quasi-static hysteresis curves. (**b**) *d* versus $H_{DC}$, calculated as a finite central difference from experimental results of [41]. Black arrows designate the direction of field change. (**c**) Phase delay ϕ versus $H_{DC}$. (**d**) Lissajous figures for different values of $H_{DC}$. Continuous lines refer to $H_{DC}=52$ kA/m, dashed lines refer to $H_{DC}=102$ kA/m, and dash-dotted lines refer to $H_{DC}=25$ kA/m.

As far as the usage of magnetostrictive polymer composites as actuators is concerned, the most interesting information is the value of the critical bias field at which the highest $d_{33}$ is observed [59]. Figure 5a presents the measurement results for isotropic MAE cylinders with an aspect ratio $\Gamma_{0}=1.2$, where the bias magnetic field $H_{DC}$ was varied, and the amplitude of field oscillations at a frequency *f* of 1.0 Hz was kept constant at $H_{AC}=25$ kA/m.

As one can see from Figure 5a, the magnetostrictive response is qualitatively different for ascending and descending bias magnetic fields. The coefficient $d_{33}$ decreased with increasing $H_{DC}$ for all weight fractions of iron content in the ascending bias field. However, in the descending bias field, local maxima of $d_{33}$ were observed at $H_{DC}=52$ kA/m. The highest value of $d_{33}\approx 140$ nm/A was found for an isotropic cylinder with 75 wt% of Fe content followed by cylinders with 70 and 80 wt% of Fe, respectively. It is important that the observed dependencies of $d_{33}$ on $H_{DC}$ were qualitatively different from those expected from the dependencies of the quasi-static effective piezomagnetic constant *d* on bias field $H_{DC}$ (Figure 5b). In the investigated range of $H_{DC}$, *d* increased with increasing $H_{DC}$ for the ascending part of the hysteresis loop, while it had a local maximum around ≈100 kA/m (black dashed line in Figure 5a,b) for the descending part of the hysteresis loop. Furthermore, $d_{33}$ was roughly one order of magnitude lower than *d*. It is obvious that the viscoelasticity of MAE materials and the non-linearity of the magnetostrictive response bring new effects, which were not observed in conventional magnetostrictive polymer composites. Additional theoretical research is required to explain our experimental results.

Due to the uncertainties of measurements, it can only be concluded that the phase lag ϕ (Figure 5c) was weakly dependent on the bias field and the dependencies of ϕ on $H_{DC}$ for cylinders with 80 and 75 wt% of Fe were about 0.1 rad higher than those for a cylinder with 70 wt% of Fe.

Figure 5d depicts Lissajous curves for the MAE cylinder with 75% of iron content and three representative values of $H_{DC}$. The features described above of the magneto-deformation response to alternating magnetic field are clearly visible there.

Note that obtained maximum values of $d_{33}∼10^{2}$ nm/A are one to two orders of magnitude higher than those in conventional magnetostrictive polymer composites, while the critical magnetic fields where the maximum of $d_{33}$ is observed are of the same order of magnitude (30–140 kA/m) [59].

**Figure 6:**
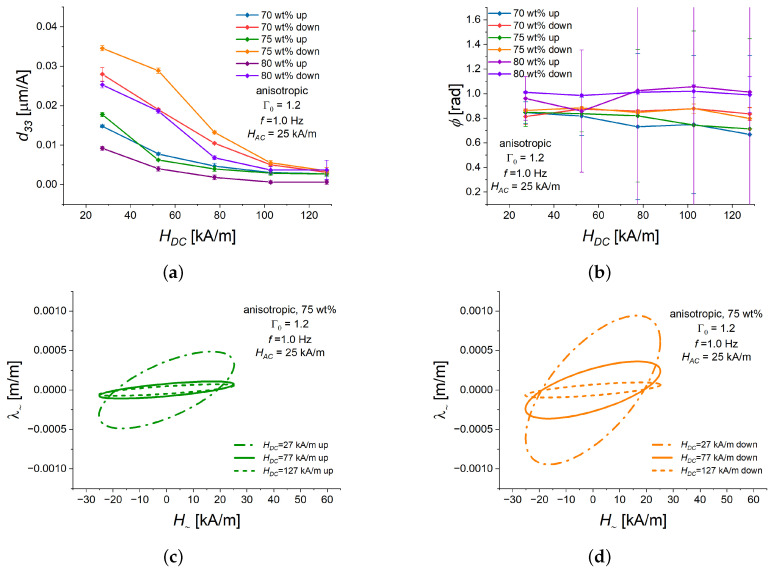
Dependencies of different characteristics of the alternating strain $\lambda_{∼}$ with a frequency *f* = 1.0 Hz on the magnitude of $H_{DC}$ for anisotropic MAE cylinders with three different weight fractions of Fe (70, 75, 80 wt%), and $\Gamma_{0}=1.2$ in ascending (“up”) or descending (“down”) bias field $H_{DC}$ of a quasi-static magnetostrictive hysteresis loop. The value of $H_{AC}$ is fixed at 25 kA/m. In all sub-figures, a specific line color refers to the same value of iron content and the same part of hysteresis loop (ascending/descending). (**a**) $d_{33}$ versus $H_{DC}$. (**b**) Phase delay ϕ versus $H_{DC}$. (**c**) Lissajous figures for different values of ascending (“up”) bias field $H_{DC}$. (**d**) Lissajous figures for different values of descending (“down”) bias field $H_{DC}$.

Anisotropic cylinders with otherwise the same material parameters exhibited much lower magnetostrictive strains [41]; also, $d_{33}$ was about 1.5-fold lower (Figure 6a). The phase lag ϕ was practically independent of $H_{DC}$ (Figure 6b). Remarkably, $d_{33}$ decreased with increasing $H_{DC}$ for both ascending and descending bias fields (Figure 6a). Local maxima were absent.

Figure 6c,d depict Lissajous curves for the anisotropic MAE cylinder with 75 wt% of Fe and three representative values of $H_{DC}$, for both ascending (Figure 6c) and descending (Figure 6d) bias fields. The features described above of the magneto-deformation response to alternating magnetic field are clearly visible there. Since the piezomagnetic response of anisotropic cylinders was lower than that of their isotropic counterparts, they will be not be considered in the following.

### 3.3. Frequency Dependence of the Alternating Strain

In our next set of experiments, we investigated the dependence of the piezomagnetic characteristics on the oscillation frequency *f* in the range between 0.1 and 2.5 Hz for a isotropic MAE cylinders with an aspect ratio $\Gamma_{0}$ of 1.2. The frequency of 2.5 Hz was the highest achievable frequency, due to the experimentally limited sampling of the alternating magnetic field. The oscillation amplitude and the bias magnetic field were held constant at $H_{AC}=25$ kA/m and $H_{DC}=77$ kA/m, respectively.

**Figure 7:**
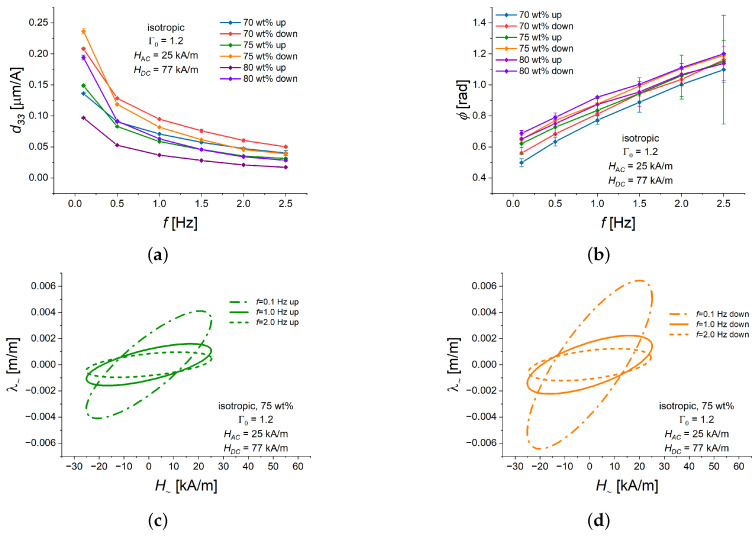
Dependencies of different characteristics of the alternating strain $\lambda_{∼}$ on the oscillation frequency *f* for isotropic MAE cylinders with three different weight fractions of Fe (70, 75, 80 wt%) and $\Gamma_{0}=1.2$ in ascending (“up”) or descending (“down”) bias field of a quasi-static magnetostrictive hysteresis loop. The value of $H_{AC}$ is fixed at 25 kA/m. The value of $H_{DC}$ is fixed at 77 kA/m. In all sub-figures, a specific line color refers to the same value of iron content and the same part of the hysteresis loop (ascending/descending). (**a**) $d_{33}$ versus *f*. (**b**) Phase delay ϕ versus *f*. (**c**) Lissajous figures for different values of frequency *f* in an ascending (“up”) bias field $H_{DC}$ of 77 kA/m. (**d**) Lissajous figures for different values of frequency *f* in a descending (“down”) bias field $H_{DC}$ of 77 kA/m.

The piezomagnetic coefficent $d_{33}$ monotonically decreased with increasing frequency *f* (Figure 7a) for each particular concentration of filling particles and the direction of bias field change. For otherwise the same experimental parameters, $d_{33}$ was somewhat higher for the descending bias field than for the ascending bias field. For each iron concentration and particular branch of hysteresis loop (up/down), the phase lag ϕ monotonically increased with increasing frequency *f* (Figure 7b). The decrease in $d_{33}$ and the increase in ϕ with increasing frequency can be attributed to the viscoelasticity of the PDMS matrix and the composite material as a whole.

Figure 7c,d depict Lissajous curves for the isotropic MAE cylinder with 75 wt% of Fe and three representative values of frequency *f* (0.1, 1.5, 2.5 Hz), both for ascending (Figure 7c) and descending (Figure 7d) bias fields, where the features described above of the magneto-deformation response to alternating magnetic field can be clearly observed.

### 3.4. Dependence of the Alternating Strain on the Aspect Ratio

**Figure 8:**
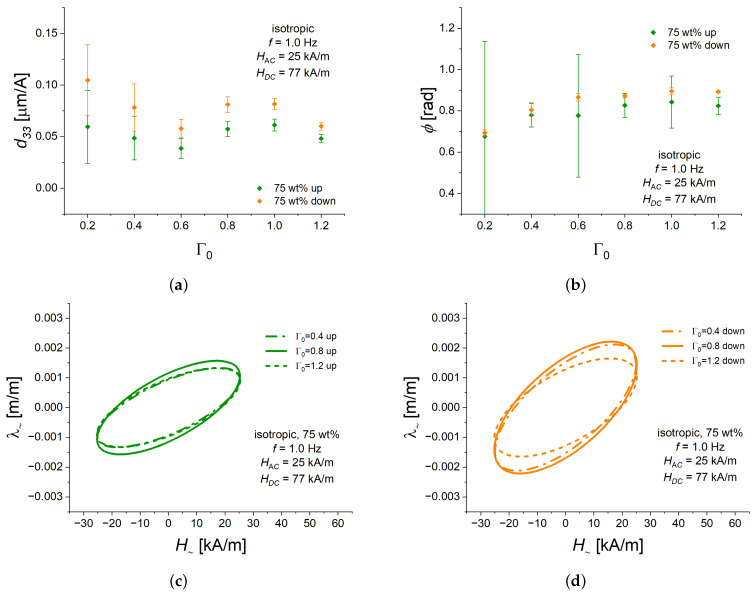
Dependencies of different characteristics of the alternating strain $\lambda_{∼}$ on the aspect ratio $\Gamma_{0}$ for isotropic MAE cylinders with 75 wt% of Fe in ascending (“up”) or descending (“down”) bias field of a quasi-static magnetostrictive hysteresis loop. The value of $H_{AC}$ is fixed at 25 kA/m. The value of $H_{DC}$ is fixed at 77 kA/m. The frequency *f* is fixed at 1.0 Hz. In all sub-figures, a specific line color refers to the same part of the hysteresis loop (ascending/descending). (**a**) $d_{33}$ versus $\Gamma_{0}$. (**b**) Phase delay ϕ versus aspect ratio $\Gamma_{0}$. (**c**) Lissajous figures for different aspect ratios $\Gamma_{0}$ in an ascending (“up”) bias field $H_{DC}$ of 77 kA/m. (**d**) Lissajous figures for different aspect ratios $\Gamma_{0}$ in a descending (“down”) bias field $H_{DC}$ of 77 kA/m. Dash-dotted lines correspond to $\Gamma_{0}=0.4$, continuous lines correspond to $\Gamma_{0}=0.8$, and dashed lines correspond to $\Gamma_{0}=1.2$.

Finally, we investigated how the piezomagnetic characteristics of isotropic MAE cylinders with 75 wt% of Fe depend on the aspect ratio $\Gamma_{0}$ (Figure 8). The amplitude of the alternating field $H_{AC}$ was equal to 25 kA/m, and the bias magnetic field $H_{DC}$ was 77 kA/m. The frequency *f* was 1.0 Hz. The primary effect of the aspect ratio should be the change in the demagnetizing factor [64]. Additionally, we have previously found that a concave dent is formed on the free circular base in magnetic fields [41]. The contribution of this dent to the apparent longitudinal strain increases with the decreasing aspect ratio [41].

Figure 8 presents the experimental results obtained. The piezomagnetic strain constant $d_{33}$ was always somewhat lower for the ascending bias field than for the descending bias field (Figure 8a), in agreement with the observations described above. In general, $d_{33}$ showed a peculiar dependence on the aspect ratio $\Gamma_{0}$ (Figure 8a), which currently have no explanation. We have verified that these results (Figure 8a) are repeatable. There seems to be a local minimum of $d_{33}$ around $\Gamma_{0}=0.6$ and a local maximum between $\Gamma_{0}=0.8$ and 1.0. An increase in the aspect ratio should lead to an increase in the internal magnetic field strength with the cylinder, which should affect both DC and AC components of the internal magnetic field. Figure 4b and Figure 5a demonstrate qualitatively different behavior of $d_{33}$ on $H_{AC}$ and $H_{DC}$, respectively. Could it be the interplay of non-linear dependencies of the coefficient $d_{33}$ on the amplitudes of the overlapping DC and AC magnetic field, which lead to the appearance of the local maximum and minimum in dependence of $d_{33}$ on the aspect ratio $\Gamma_{0}$? This question has to be investigated in detail in future works.

Within the uncertainty of measurements, the phase lag ϕ was independent of the aspect ratio $\Gamma_{0}$ for both ascending and descending bias magnetic fields (Figure 8b).

Figure 8c,d provide the Lissajous figures of the alternating strain versus the alternating magnetic field for three selected values of the aspect ratio $\Gamma_{0}$. They present the results of Figure 8a,b in an alternative form.

## 4. Conclusions and Outlook

To summarize, we presented a comprehensive experimental study of the deformation response of MAE cylinders to time-harmonic magnetic fields in the vicinity of different operating points (bias magnetic fields). To the best of our knowledge, such an investigation was performed for the first time. It was found that the behavior of MAEs was different from conventional magnetostrictive polymer composites in many respects. The phenomenon resembles piezomagnetism in the sense that there are positive and negative half-cycles of alternating strain in response to a time-harmonic magnetic field, but there is a significant phase delay (≈0.6–1.0 rad) between the alternating magnetic field and alternating strain. Obviously, if time-harmonic quantities $H_{AC},\lambda_{AC}$ are written in the form of a phasor, $d_{33}$ should be a complex number. The strain response to an oscillating magnetic field also involves a transient component (Figure 3d). The dependence of the piezomagnetic strain coefficient $d_{33}$ on the amplitude of the alternating magnetic field $H_{AC}$ was non-linear in the investigated range of $H_{AC}$ (Figure 4b). The obtained values of $d_{33}$ were one to two orders of magnitude higher than in conventional magnetostrictive polymer composites (Figure 4b and Figure 5a). At a particular value of the bias field $H_{DC}$, $d_{33}$ was higher when this value was reached at the descending part of the magnetostrictive hysteresis loop in comparison with the ascending part of the loop. In the investigated frequency range (0.1–2.5 Hz), the piezomagnetic strain coefficient $d_{33}$ decreased with an increasing frequency *f* of alternating magnetic field.

It has been found that the strain response of MAE cylinders to a time-harmonic magnetic field superimposed on a biased magnetic is rather complex. It has several intriguing features not observed in conventional magnetostrictive polymer composites. It became also obvious that dynamic behavior of MAE cylinders cannot be simply derived from the quasi-static measurements. Unfortunately, there are currently no theories available to explain observed experimental dependencies. Given that MAE materials are considered to be promising as actuators in soft robotics [29,30], we hope that our experimental findings will encourage theoreticians to develop novel dynamic models incorporating non-linear viscoelasticity and inertia [50] for describing macroscopic deformations of MAE-based functional elements.

## Figures and Tables

**Figure A1:**
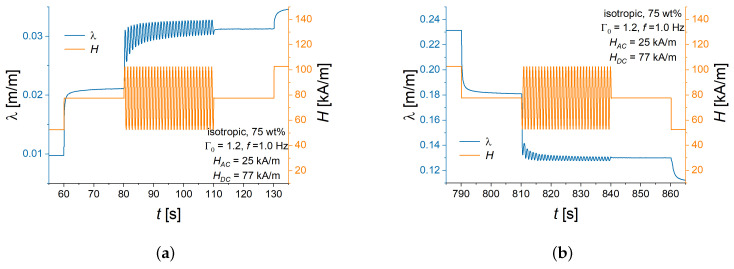
Time dependencies of the longitudinal strain (blue curve) for harmonic magnetic field modulations (orange curve) when the DC magnetic field is increasing (**a**) and when it is decreasing (**b**). Parameters of the sample and measurement conditions are given within the figures.

## Data Availability

The data supporting the findings of this paper ara available in Zenodo https://doi.org/10.5281/zenodo.13834054 (accessed on 24 September 2024).

## Appendix A

Figure A1a shows in detail the transient behavior of the longitudinal strain when the DC magnetic field strength is increased from 52 kA/m to 77 kA/m and then overlapped by a sinusoidal magnetic field. Figure A1b presents in detail the transient behavior of the longitudinal strain when the DC magnetic field strength is decreased from 102 kA/m to 77 kA/m and then overlapped by a sinusoidal magnetic field. In both figures, the following step-like change in the external magnetic field is visible as well. For the sake of detailed presentation, the images for the calculation of the longitudinal strain were taken at 18 FPS. Notice that after the modulating AC magnetic field is switched off, the resulting longitudinal strain seems to be practically time independent, and it is not equal to the steady-state longitudinal strain in the DC field before the modulating AC magnetic field is switched on.

## Appendix B

Figure A2 presents enlarged views of the two curves in Figure 3d, separately.
